# The impact of dysfunctional variants of ABCG2 on hyperuricemia and gout in pediatric-onset patients

**DOI:** 10.1186/s13075-019-1860-8

**Published:** 2019-03-20

**Authors:** Blanka Stiburkova, Katerina Pavelcova, Marketa Pavlikova, Pavel Ješina, Karel Pavelka

**Affiliations:** 1 Institute of Rheumatology, Na Slupi 4, 128 50 Prague 2, Czech Republic; 2 Department of Rheumatology, First Faculty of Medicine, Charles University, Prague, Czech Republic; 3 Department of Pediatrics and Adolescent Medicine, First Faculty of Medicine, Charles University and General University Hospital in Prague, Prague, Czech Republic

**Keywords:** Gout, Hyperuricemia, Urate transport, ABCG2

## Abstract

**Background:**

ABCG2 is a high-capacity urate transporter that plays a crucial role in renal urate overload and extra-renal urate underexcretion. Previous studies have suggested an association between hyperuricemia and gout susceptibility relative to dysfunctional ABCG2 variants, with rs2231142 (Q141K) being the most common. In this study, we analyzed the *ABCG2* gene in a hyperuricemia and gout cohort focusing on patients with pediatric-onset, i.e., before 18 years of age.

**Method:**

The cohort was recruited from the Czech Republic (*n* = 234) and consisted of 58 primary hyperuricemia and 176 gout patients, with a focus on pediatric-onset patients (*n* = 31, 17 hyperuricemia/14 gouts); 115 normouricemic controls were used for comparison. We amplified, sequenced, and analyzed 15 *ABCG2* exons. The chi-square goodness-of-fit test was used to compare minor allele frequencies (MAF), and the log-rank test was used to compare empirical distribution functions.

**Results:**

In the pediatric-onset cohort, two common (p.V12M, p.Q141K) and three very rare (p.K360del, p.T421A, p.T434M) allelic ABCG2 variants were detected. The MAF of p.Q141K was 38.7% compared to adult-onset MAF 21.2% (OR = 2.4, *P* = 0.005), to the normouricemic controls cohort MAF 8.5% (OR = 6.8, *P* <  0.0001), and to the European population MAF 9.4% (OR = 5.7, *P* <  0.0001). The MAF was greatly elevated not only among pediatric-onset gout patients (42.9%) but also among patients with hyperuricemia (35.3%). Most (74%) of the pediatric-onset patients had affected family members (61% were first-degree relatives).

**Conclusion:**

Our results show that genetic factors affecting ABCG2 function should be routinely considered in a hyperuricemia/gout diagnosis, especially in pediatric-onset patients. Genotyping of *ABCG2* is essential for risk estimation of gout/hyperuricemia in patients with very early-onset and/or a family history.

## Background

Serum urate concentration is a complex phenotype influenced by both genetic and environmental factors, as well as their interactions. Hyperuricemia results from an imbalance between endogenous production and excretion of urate. The most common mechanism leading to hyperuricemia is decreased excretion of urate. Hyperuricemia is a central feature in the pathogenesis of gout. Gout is a metabolic disorder caused by an inflammatory reaction to the deposit of urate crystals in joints and soft tissues. The disorder progresses through several degrees, and chronic hyperuricemia is a necessary condition for gout to develop. Prevalence of gout is higher in men [1], and women with gout are more likely to be older (link with menopause) [2], have co-morbidities, and be on diuretics compared with men with gout [3, 4]. Gout usually occurs between the fourth and sixth decade of life. Pediatric-onset of hyperuricemia and gout in clinical practice is rare and suggestive of a genetic disorder as PRPS1 superactivity [5] and hypoxanthine-guanine phosphoribosyltransferase deficiency [6], especially when a strong family history is obtained.

Serum urate (SU) concentrations are heritable (0.38–0.63) [7–9], which is consistent with a significant genetic component. Over the past decade, genome-wide association studies (GWAS) and meta-analyses have led to an increase in our knowledge of the common genetic variants that influence SU concentrations. To date over 30 common sequence variants, which can affect hyperuricemia/gout have been revealed, most of which are in urate transporters [10, 11]. Urate transport is a complex process involving several transmembrane proteins that provide reabsorption (e.g., URAT1, GLUT9) and secretion (ABCG2). They are located on the apical and basolateral membrane of proximal tubule cells. ABCG2 also plays a significant role in regulating uric acid transport in the gastrointestinal tract [12]. The genetic predisposition to hyperuricemia is evidenced by monogenic diseases and population-based studies [13]. However, detailed knowledge of the degree to which genetic variants predict SU concentrations remains limited.

ABCG2 is a high-capacity urate transporter that plays a crucial role in renal urate overload and extra-renal urate underexcretion. Many previous studies have indicated that the common dysfunctional variants rs72552713 (p.Q126X) and rs2231142 (p.Q141K) increase the risk of gout and hyperuricemia, significantly influence the age of onset of gout, and are highly associated with a familial gout history [14, 15]. Variant p.Q126X, a common variant in the Japanese population, is a rare variant in European and African-American populations, whereas p.Q141K is a common variant in all these populations [16]. The ABCG2 population-attributable percent risk for hyperuricemia has been reported to be 29.2%, which is much higher than those with more typical environmental risks, i.e., BMI ≥ 25.0 (18.7%), heavy drinking (15.4%), and age (≥ 60 years old, 5.74%) [17]. In a GWAS of clinically defined gout, the ABCG2 locus showed the most significant association with gout susceptibility [11, 18, 19]. These findings indicate that common variants of ABCG2 are extremely important in gout pathogenesis. However, some variants of increased penetrance that are associated with gout are population specific and/or uncommon. Approximately 80% of Japanese patients with gout have been reported to possess either the p.Q126X or p.Q141K variant of ABCG2 [20], and these variants increased the risk of gout conferring an OR of more than 3 [14]. On the other hand, the 19 rare non-synonymous variants of ABCG2 identified in Japanese gout cohort [21] were not shared among the population samples that were tested in Czech gout cohort where eight rare non-synonymous ABCG2 variants were identified [15, 21]. These populations, with a specific distribution of dysfunctional ABCG2 variants, thus increase the risk of gout.

In our previous study, we analyzed the *ABCG2* gene [15] in a cohort of 145 subjects with gout. Our results showed a higher minor allele frequency of the p.Q141K variant in the gout patients (0.23) compared with the European-origin population (0.09) and were significantly more common among gout patients than among normouricemic controls (odds ratio = 3.26, *P* <  0.0001). Our analysis shows also an apparent shift in proportions of patients with non-synonymous alleles who are over-represented in an earlier age of onset categories and under-represented in older age of onset categories (χ2-test for the trend in proportions, *P* = 0.010). Such over-representation merited a detailed exploration. We therefore expanded the cohort by including both newly recruited gout patients (five pediatric-onset) and the hyperuricemic patients (17 pediatric-onset).

Until now, no study of individual variants of the ABCG2 transporter, using a well-characterized pediatric-onset cohort suffering from primary hyperuricemia/gout (i.e., considering clinical data on purine metabolism, the occurrence of associated diseases, familiar anamnesis, medication), has been performed. In this study, we analyzed the *ABCG2* gene in a Czech hyperuricemia and gout cohort focusing on pediatric-onset (before 18 years of age) patients. Our data show, for the first time, that ABCG2 dysfunction is a strong independent risk for pediatric-onset of hyperuricemia and gout.

## Methods

The definition of hyperuricemia was as follows: (1) men > 420 μmol/l on two repeated measurements and (2) women and children under 15 years > 360 μmol/l on two repeated measurements, taken at least 4 weeks apart. Gouty arthritis was diagnosed according to the American College of Rheumatology criteria, i.e., (1) the presence of sodium urate crystals seen in the synovial fluid using a polarized microscope or (2) subjects meet 6 of 12 clinical criteria [22].

A cohort of 145 previously described gout subjects (9 with pediatric-onset) was enlarged to 176 gout patients (5 more with pediatric-onset), and a group of 58 hyperuricemic patients (17 pediatric-onset) was added. In total, 234 hyperuricemic or gout patients were recruited, 31 with a pediatric-onset (22 newly recruited patients). The age of ascertainment (hyperuricemia) and onset (gout) was determined as the age of laboratory diagnosis in case of asymptomatic hyperuricemia, or as the first symptoms of gout. For the sake of shortness, the term “onset” is used for both situations.

Asymptomatic hyperuricemic patients with pediatric-onset were identified through a random finding in a routine laboratory examination (e.g., for infectious diseases). In one case, the patient was ascertained based on a positive family history.

The pediatric-onset group of 31 patients from 30 families (one pair of siblings) consists of two separate sets. The first part consisted of 15 pediatric patients with hyperuricemia (10 subjects) and gout (5 subjects), mostly from the Department of Pediatrics and Adolescent Medicine, which includes the Metabolic Center (the only one in the Czech Republic involved in the Metabolic European Reference Network MetabERN). This set is complemented with 16 adult patients with pediatric-onset of hyperuricemia (7 subjects) and gout (9 subjects) from the Institute of Rheumatology (super-conciliar institute for the Czech Republic). All patients were residents of the Czech Republic, Central-European population, with no history or signs of renal diseases.

**Fig. 1:**
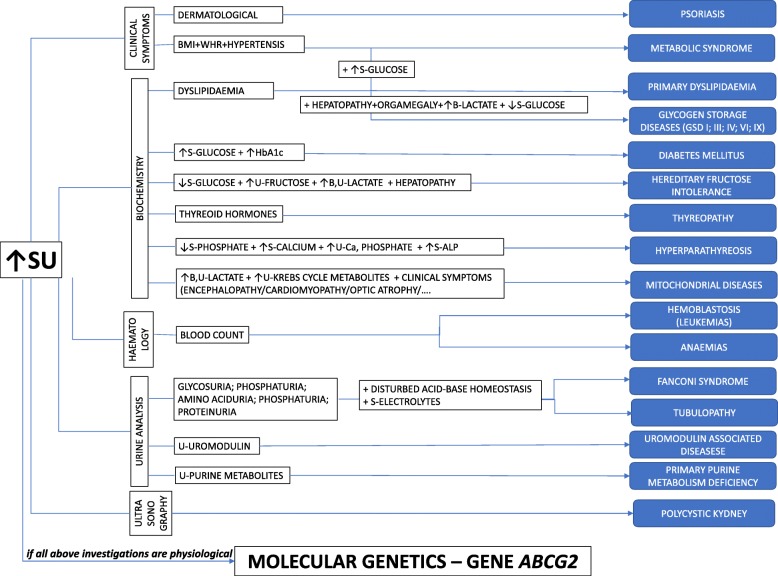
Differential diagnostic algorithm in a pediatric-onset patient with hyperuricemia. (BMI body mass index, WHR wait to hip ratio, HbA1c glycated hemoglobin, Ca calcium, ALP alkaline phosphatase, SU serum urate, GSD glycogen storage disorders, B blood, U urine, S serum)

To explore the cause of hyperuricemia and gout, we performed a detailed metabolic investigation. The biochemical tests were performed using morning urine samples; 24-h urine collections were not available. Patients suffering from secondary gout and other purine metabolic disorders associated with pathological concentrations of SU (such as the reduced activity of hypoxanthine-guanine phosphoribosyltransferase and superactivity of phosphoribosyl pyrophosphate synthetase 1, i.e., resulting in increased excretion of xanthine and hypoxanthine in urine) were excluded. Pediatric subjects were specifically screened for kidney disorders (Fanconi syndrome and uromodulin-associated disorders) and for metabolic genetic disorders (glycogen storage disease, hereditary fructose intolerance, and mitochondrial disorders). Patients with such disorders were excluded from the study. The diagnostic algorithm and appropriate examinations are summarized in Fig. 1.

The analysis of *ABCG2* was performed from genomic DNA, as reported previously [8]. All tests were performed in accordance with standards set by the institutional ethics committees (no. 6181/2015). All statistical analyses were performed in the statistical language and environment R, v. 3.5. The Wilcoxon two-sample test and the Fisher exact test were used to compare group characteristics, the *χ*^2^ goodness-of-fit test was used to compare minor allele frequencies, and the log-rank test was used to compare empirical distribution functions. The role of possible confounders was checked through the Mantel-Haenszel test and logistic regression.

## Results

In the cohort of 234 Caucasians suffering from hyperuricemia (*N* = 58) or primary gout (*N* = 176), we focused on 31 individuals with pediatric-onset of either hyperuricemia or gout. The main characteristics of both pediatric-onset and adult-onset groups are summarized in Table 1. Of the 31 pediatric-onset subjects, 19 non-consanguinity patients had at least of one non-synonymous allelic variant in the coding region of the *ABCG2* gene. Their main characteristics are summarized in Table 2.

**Table 1:** Demographic, biochemical, and genetic characteristics of pediatric-onset (*N* = 31) and adult-onset (*N* = 203) cohorts

	Pediatric-onset (*N* = 31)		Adult-onset (*N* = 203)		Fisher’s test *P* value
	***N***	**%**	***N***	**%**	
Sex M/F	26/5	83.9/16.1	174/29	85.7/14.3	0.786
Hyperuricemia/primary gout	17/14	58.1/41.9	41/162	79.8/21.2	< 0.0001
Familial occurrence	23	74.2	64	31.5	< 0.0001
Familial occurrence,					
1st degree	19	61.3	Not specified		–
2nd degree	4	12.9			
No treatment	15	48.4	36	17.7	< 0.0001
Allopurinol treatment	12	38.7	154	75.9	
Febuxostat treatment	4	12.9	13	6.4	
rs2231142					
GG	14	45.2	124	61.1	0.001
GT	10	32.3	72	35.5	
TT	7	22.6	7	3.4	
rs2231142, MAF	24	38.7	86	21.2	0.005
rs2231137, MAF **	1	1.6	7	1.7	1.000
rs769734146, MAF	1	1.6	1	0.2	0.623
rs750972998, MAF	1	1.6	0	0.0	0.278
rs199854112, MAF	1	1.6	0	0.0	0.278
	Median (IQR)	Range	Median (IQR)	Range	Wilcoxon’s test *P* value
Age of onset, years	15.0 (4.0)	1–18	43.5 (24.2)	18–84	< 0.0001
Age now, years	19.0 (19.5)	3–59	55.0 (22.0)	19–90	< 0.0001
BMI now	25.1 (7.6)	16.0–41.0	29.0 (5.1)	19.5–50.0	< 0.0001
Max recorded SU, μmol/l (*N* = 34/155) #	522.0 (144.0)	314–796	481.0 (101.0)	252–770	0.021
SU on treatment, μmol/l (*N* = 17/176) #	419.0 (96.0)	300–608	371.5 (132.5)	252–770	0.091
FE-U on treatment (*N* = 15/173) #	3.2 (1.7)	1.6–5.5	3.2 (1.7)	0.9–14.3	0.403
Treatment dose, mg * (*N* = 15/168)	100 (200)	80–500	200 (200)	0–800	0.181

#For some parameters, there were missing data; in case missing data amounted to 5% or more, the real *N* is mentioned in parentheses in the form *N*_adolescent_/*N*_adult_

*Febuxostat dose was recomputed so that 40 mg febuxostat = 300 mg allopurinol

**Of the functional ABCG2 variants explored in [15], the five mentioned in the table were present among adolescent-onset patients. The variants rs372192400, rs753759474, rs752626614, and p.S476P (not annotated) had MAF 0.0025 among adult-onset patients, and rs34783571 had MAF 0.0049 among adult-onset patients. Neither of them appeared among adolescent-onset patients (*P* values of the test for difference were equal to 1.000)

**Table 2:** Demographic, biochemical, and genetic characteristics of pediatric-onset patients with non-synonymous ABCG2 variants (*N* = 19) and reference sequence of ABCG2 (*N* = 12)

Age of onset/measurement	Sex	Hyperuricemia	Gout	ABCG2	Familiarity	BMI	Metabolic syndrome	SU	FE-U	U-U	u-Hypoxanthine	u-Xanthine
Year		Evidence		aa	Affected family members		Number of criteria				Ref. ranges ≤ 30	Ref. ranges ≤ 25
								μmol/L	%	mmol/mol creatinine		
17/21	M	Yes	No	p.[Q141K];[Q141K]	–	23	0	507	*4.7	0.2	3.5	2.1
16/31	M	Yes	Yes	p.[Q141K];[Q141=]	Pat. grandfather	39**	2	796	*2.0	0.16	2.5	9.8
18/40	F	Yes	Yes	p.[V12M];[V12=]	Father	21	0	546	*2.6	0.17	4.1	14.2
18/59	M	Yes	Yes	p.[Q141K];[Q141=]	Mother	30**	1	492	5.2	0.33	15.4	10.2
16/17	M	Yes	Yes	p.[Q141K];[Q141=]	Two brothers, pat. grandfather	22	0	540	*2.5	0.2	11.7	8.6
14/15	F	Yes	No	p.(Q141K)(;)(T434M)	–	24	0	495	*3.2	0.28	2.1	3.2
8/54	M	Yes	Yes	p.[Q141K];[Q141K]	Brother, father, pat. grandfather	41**	2	514	5	0.31	2.9	2.3
18/37	M	Yes	Yes	p.(Q141K)(;)(K360del)	–	30**	2	627	5	0.3	5.5	Under limit
13/14	M	Yes	Yes	p.[Q141K];[Q141=]	Father, pat. grandfather	24	1	621	*4.8	0.48	13.5	3.6
15/15	M	Yes	No	p.[Q141K];[Q141K]	Father	18	0	522	*3.9	0.3	13.5	8.2
15/39	M	Yes	Yes	p.[Q141K];[Q141=]	n/a	30**	2	670	*2.5	*0.09	24.3ª	49.9*ª
17/18	M	Yes	No	p.[T421A];[T421=]	Father	24	1	420	*3.9	0.15	2.4	Under limit
16/18	M	Yes	Yes	p.[Q141K];[Q141K]	–	27*	2	655	*4.3	0.35	1.2	2
6/11	F	Yes	No	p.[Q141K];[Q141=]	Mother, mat. grandmother	26*	1	430	*2.1	0.19	6.9	6.6
18/19	M	Yes	No	p.[Q141K];[Q141=]	Mother, mat. uncle	25	1	439	*4.9	0.27	5.4	2.9
12/12	F	Yes	No	p.[Q141K];[Q141=]	Brother, mother	30**	1	473	*2.3	0.29	5.4	6.5
14/14	M	Yes	No	p.[Q141K];[Q141K]	Father	22	0	465	6	0.31	15.3	27.5
13/14	M	Yes	Yes	p.[Q141K];[Q141K]	Mother	21	NA	506	*3.0	0.23	3.2	3.1
18/19	M	Yes	No	p.[Q141K];[Q141K]	Pat. grandmother	26*	1	730	5.5	0.35	3.4	2.8
11/20	M	Yes	No	No allelic variants	Mother	28*	0	662	*2.6	0.18	17	11.2
14/19	M	Yes	No	No allelic variants	–	27*	2	631	*2.0	0.17	13.1	18.4
1/3	F	Yes	No	No allelic variants	Mother	16	0	522	*2.6	0.47	9.4	8.7
15/21	M	Yes	No	No allelic variants	Pat. grandmother	35**	1	524	*3.8	0.17	20.1^b^	26.1*^b^
10/11	M	Yes	No	No allelic variants	Brother, father	20	0	384	*4.0	0.56	79*^a^	120*^a^
13/14	M	Yes	No	No allelic variants	Brother, father	24	0	435	*3.7	0.23	7.8	4.7
12/57	M	Yes	Yes	No allelic variants	Son, father, pat. grandfather	37**	2	442	*3.5	0.18	2.9	1.1
13/24	M	Yes	No	No allelic variants	Father, pat. grandfather and great-gr.	24	0	576	5.1	0.32	2	Under limit
17/18	M	Yes	Yes	No allelic variants	–	25	0	436	7.3	0.28	10	2.5
18/48	M	Yes	Yes	No allelic variants	–	26*	1	487	7.2	0.44	9.9	2.6
13/15	M	Yes	No	No allelic variants	Pat. and mat. grandfather	18	0	630	*2.9	0.46	3	4.3
18/45	M	Yes	Yes	No allelic variants	Brother, father	30**	3	610	5.0	0.35	1.7	1.2

*>< ref. range; ^a^measurement with febuxostat therapy 80 mg/per day; ^b^measurement with allopurinol therapy 150 mg/per day; SU < 15 years and female 120–340 μmol/l, male 120–416 μmol/l; FE-U < 13 years 5–20%, male 5–12%, female 5–15%; U-U < 15 years 0.1–1.0 mmol/mol creatinine, > 15 years 0.1–0.8 mmol/mol creatinine

Although pediatric-onset patients were consecutively included in the study, and even though they often did not show renal function impairment and were not taking medications known to change renal handling of uric acid (such as diuretics, aspirin, cyclosporine, pyrazinamide), the excretion fraction of urate (FE-U) was under the reference range in 14 of 19 patients with allelic *ABCG2* variants, while another two patients were at the lower border of the reference range. Only one patient has decreased urate levels in their urine (the lower limit was ≤ 0.1 mmol/L of UA per mmol/L of creatinine, which represents the lower 2.5th percentile of the entire reference range). In terms of percentiles, 7 patients were below the 5th percentile of the reference range (≤ 0.2 mmol/L of urate per mmol/L of creatinine), 13 patients were below the 20th percentile (≤ 0.3 mmol/L of urate per mmol/L of creatinine), and 18 patients were below the 40th percentile (≤ 0.4 mmol/L of urate per mmol/L of creatinine).

**Fig. 2:**
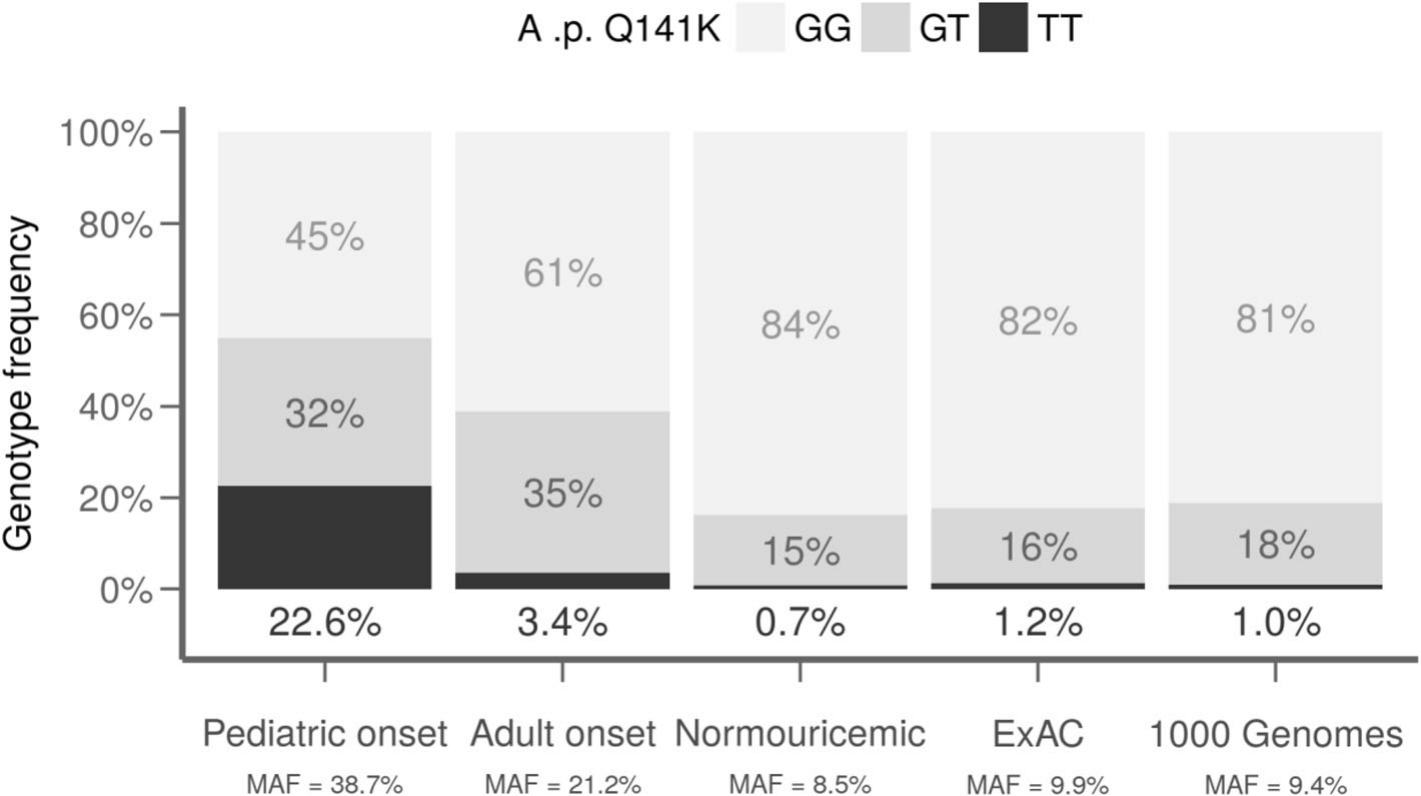
Genotype frequency of p.Q141K in a pediatric-onset cohort with hyperuricemia/gout (31 subjects, MAF = 38.7%) compared to an adult-onset hyperuricemia/gout (203 subjects, MAF = 21.2%), and a normouricemic control cohort (115 subjects, MAF = 8.5%), and data from the ExAC and 1000 Genome databases

The analysis of *ABCG2* in the pediatric-onset cohort revealed two common non-synonymous variants (rs2231137 (p.V12M), rs2231142 (p.Q141K)) and three rare heterozygous non-synonymous variants (in-frame deletion rs750972998 (p.K360del), missense variant rs199854112 (p.T421A), and rs769734146 (p.T434M)). Seven of the 31 pediatric-onset patients were homozygous, and 10 were heterozygous for p.Q141K. This makes the minor allele frequency (MAF) of p.Q141K 38.7% compared to (1) the adult-onset MAF = 21.2% (OR = 2.4, *P* = 0.005), (2) 115 normouricemic controls MAF = 8.5% (OR = 6.8, *P* <  0.0001 [15]), and (3) the European population (source 1000 Genomes Project Phase 3) MAF = 9.4% (OR = 5.7, *P* <  0.0001, Fig. 2). To add to the picture, the MAF of p.Q141K was 35.3% (4 homozygotes, 4 heterozygotes) among the 17 pediatric-onset hyperuricemic patients and 42.9% (3 homozygotes, 6 heterozygotes) among the 14 pediatric-onset gout patients; meaning that a high frequency of p.Q141K was detected not only among symptomatic gout patients but among asymptomatic hyperuricemic patients as well.

As for other non-synonymous variants, one pediatric-onset gout patient was heterozygous for p.Q141K, and the rare p.K360del variant and another gout patient were heterozygous for the rare p.T421A variant. One hyperuricemic pediatric-onset patient was heterozygous for p.Q141K and the rare p.T434M variant. Those rare variants were not found among the adult-onset patients. One pediatric-onset hyperuricemic patient was heterozygous for p.V12M with a MAF of 1.6%, which was similar to the adult-onset MAF of 1.7%.

The in-frame three nucleotide deletion p.K360del (European MAF = 0.007) was located in the intracellular membrane-spanning domain. Variants p.T421A (European MAF <  0.0001) and p.T434M (European MAF <  0.0001) were located in the transmembrane domain 2. The in silico analysis (PROVEAN and SIFT) predicted a neutral impact for p.K360del and p.T421A and a deleterious impact for p.T434M.

**Fig. 3:**
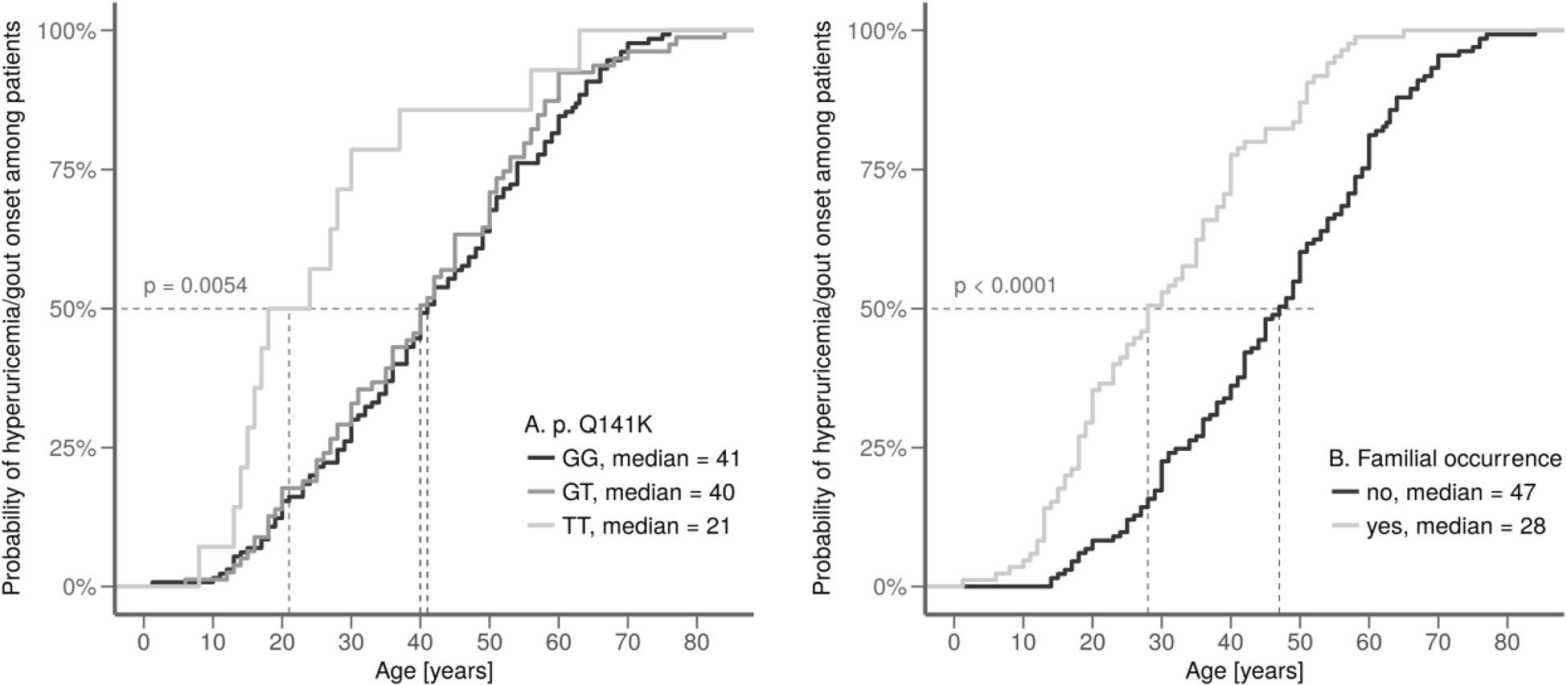
**a** The influence of p.Q141K: homozygotes develop hyperuricemia very early compared to heterozygotes and wild-type homozygotes. **b** The existence of familial hyperuricemia/gout shifts the age of onset towards earlier ages in the whole set

In our pediatric-onset cohort, the mean age of hyperuricemia onset was 13.0 years, for gout the onset it was 15.4 years. Figure 3a shows the major role of p.Q141K homozygosity in the onset of gout/hyperuricemia from another point of view, while the median age of hyperuricemia/gout onset was around 40 years for heterozygotes and wild-type homozygotes, it was significantly lower, i.e., 21 years, for p.Q141K homozygotes (*P* = 0.005).

Among the 31 pediatric-onset patients, we found that 23 (74%) had affected family members (in 19 cases, 61% were first degree relatives). This was more than twice that seen in the adult-onset group (31%, *P* <  0.0001, Table 1). The trend was even stronger for hyperuricemic patients (82%) compared to (62%) for adolescent-onset gout patients. Alternatively, while patients without a family history of hyperuricemia/gout had a median onset age of 47 years, patients with affected family members had a median onset age of 28 years (*P* <  0.0001, Fig. 3b).

## Discussion

Elevated urate concentration is central to the pathogenesis of gout. Renal underexcretion of urate, due to the dysfunction of the ABCG2 high-capacity urate exporter, is a major contributor to hyperuricemia. This study identified a high frequency of ABCG2 variants, common and rare, in a cohort of pediatric-onset primary hyperuricemia and gout patients.

The common dysfunctional variant, p.Q141K, results in a 53% reduction in urate transport and has been reported to be a major genetic cause of gout in the European population [23]. The second most common variant in European, p.V12M (rs2231137, MAF 0.06), does not appear to affect urate transport [13] and a previously reported meta-analysis indicates that p.V12M has a gout protective effect (OR = 0.73, *P* <  0.0001) [15]. The contribution of ABCG2 variants in predicting primary hyperuricemia/gout seems to be limited mainly to the absence of a functional characterization of rare variants. However, the allelic variants can be easily analyzed, and their identification can suggest a hyperuricemia/gout risk prognosis. Functional characterizations of the rare variants p.K360del, p.T421A, and p.T434M are not available. However, an in silico analysis predicts a neutral impact for p.K360del and p.T421A and damaging impact for p.T434M. Future function studies regarding the impact of those variants are necessary to determine the correlation between functional studies and scores from prediction algorithms. For example, this relationship does not exist for the most frequent dysfunctional variant p.Q141K: a number of functional analyses in *Xenopus laevis* oocytes and HEK cells showed significantly decreased protein expression and function. However, p.Q141K is classified as tolerated by PolyPhen, SIFT, Provean, Mutation Taster, MutPred, and Human Splicing Finder software.

Our data showed that ABCG2 dysfunction was a strong independent risk for pediatric-onset hyperuricemia/gout: the MAF of p.Q141K was 38.7% compared to adult-onset with a MAF of 21.2%, compared to the normouricemic controls cohort with a MAF of 8.5%, and to the European population with a MAF of 9.4%. Compared to the whole hyperuricemia/gout cohort, there is an apparent shift in the proportion of patients with non-synonymous alleles who are over-represented in pediatric-onset patients (not only for gout but also for hyperuricemia) and under-represented in older age of onset categories. A hereditary component for pediatric-onset hyperuricemia/gout was further supported by the observation of a statistically significant association of familial hyperuricemia/gout in the pediatric-onset cohort: 74% in the pediatric-onset cohort and 33% in adult-onset (*P* <  0.0001). Taken together, our data suggested that ABCG2 genetic variants have a strong impact on the progression of hyperuricemia and gout in pediatric-onset patients and imply the importance of ABCG2 genotyping for the screening of high-risk individuals.

In summary, our findings confirmed a heritability component for hyperuricemia and gout. This relationship was recently demonstrated in a cohort of asymptomatic male offspring of parents with gout, in which the male offspring had a significantly higher frequency of hyperuricemia, urate under-excretion, and prevalence of monosodium urate crystal deposits [24]. A positive family history is an important diagnostic clue; however, it may be absent in de novo mutations and where hyperuricemia/gout was either not diagnosed or not communicated to the rest of the family.

Detailed studies of SU in children are very rare. The first GWAS of SU was performed in the Viva La Familia Study [25]. This study found (1) SU concentrations were significantly heritable and (2) strong associations with genetic variants of the *SLC2A9* urate transporter. However, this GWAS did not extend the association of variants in the ABCG2 genetic locus with SU concentrations to children in a family-based study. Our analysis of *SLC2A9* coding regions in a pediatric-onset cohort revealed three synonymous variants in exon regions and three common heterozygous non-synonymous variants rs2276961 (p.G25R), rs3733591 (p.R294H), and rs2280205 (p.P350L). The MAF of these variants were similar to those in the adult-onset hyperuricemia/gout cohort and in the European population. Moreover, our previous study, which used association analysis together with functional and immunohistochemical characterization of these variants identified in the adult population, did not show any influence of these allelic variants on expression, subcellular localization, or urate uptake of GLUT9 transporters [26]. These different findings can partly be attributed to the population substructure and sample size considering that the majority of the GWAS with a strong association between ABCG2 and hyperuricemia/gout were conducted in European or Asian descent populations.

ABCG2 transporters are expressed on the apical epithelium membrane of the small intestines and colon, in erythrocyte membranes, apical membranes of kidney proximal tubular cells, and the canalicular membrane of hepatocytes [27]. Reduced and loss-of-function ABCG2 variants are associated with significantly decreased extra-renal clearance of urate [28]. The animal model of Abcg2-knockout mice showed increased serum urate and renal urate excretion and decreased intestinal urate excretion [12]. Moreover, a significant association between the common variant p.Q141K and an increased risk of a poor response to allopurinol has been described [29–31].

Data about the parameters of renal handling of urate in hyperuricemia/gout patients are not frequently reported in the literature. Perez-Ruiz et al. 2002 reported that renal underexcretion is the main mechanism for the development of primary hyperuricemia in gout, but even patients showing apparently high 24-h urate output showed lower urate clearance than controls, indicating that relative, low-grade underexcretion of urate was at work [32].

Our data in a pediatric-onset cohort with allelic ABCG2 variants showed a decrease in FE-U together with decreased urinary levels of urate. The published data about the relationship between ABCG2 dysfunction and the fractional excretion of urate are inconsistent. Matsuo et al. [18] observed an increase in FE-U associated with ABCG2 dysfunction. In contrast, Köttgen et al. [10] showed that, while the ABCG2 p.Q141K allele raised SU by 13 μmol/L (0.217 mg/dL) per risk allele in Europeans, there was a small decrease of 0.076% in FE-U (*P* = 9.8 × 10^−3^). Kannangara et al. [33] found no association between the *ABCG2* genotype and FE-U (*r* = 0.02, *P* = 0.83). Overall, these data indicate the very little clinical effect of the p.Q141K polymorphism on FE-U. These observations are in compliance with the concept of Ichida et al. [12] that the currently considered “overproduction type” hyperuricemia should be renamed to “renal overload type,” comprising two subtypes: “extra-renal urate underexcretion” and genuine “urate overproduction.” The common dysfunction of ABCG2 thus can cause a decrease of urate excretion via the extra-renal pathway rather than the renal pathway.

Hyperuricemia is an important risk factor for gout and has significant associations with several other conditions including hypertension, cardiovascular disease, and chronic kidney disease. The potential role of urate-lowering therapy, in the management of these “non-gout diseases,” has been raised [34]. However, according to the American College of Rheumatology guidelines, therapy is not recommended for people with asymptomatic hyperuricemia. Our data showed, for the first time, that ABCG2 dysfunction is a strong independent risk in pediatric-onset hyperuricemia and gout where other factors that appear in adulthood, such as alcohol consumption, diuretic use, and increase in BMI, may further increase the risk of elevated serum urate levels. The high frequency of p.Q141K, which was detected not only among pediatric-onset gout patients but also among asymptomatic hyperuricemic pediatric-onset patients, confirmed the powerful effect of ABCG2 dysfunction on the early development of hyperuricemia and gout. Further studies into the progress of pediatric-onset hyperuricemia and its development into gout later in adult life are badly needed. Taken together, our findings strongly suggest the need for a discussion about the potential benefits of urate-lowering therapy after a diagnosis of hyperuricemia in pediatric-onset patients with ABCG2 dysfunction.

Our study has two important strengths: (1) our cohort included pediatric-onset hyperuricemia and gout patients, which are relatively rare, and includes detailed biochemical characteristics and family information and (2) we controlled for several potential confounders, such as kidney diseases and metabolic diseases that might have influenced measured SU concentrations. Limitations of this study must also be acknowledged: (1) the size of the studied group may not have been sufficiently large, and it is possible that some very rare ABCG2 and SLC2A9 associated variants may have gone undetected and (2) the number of frequent genetic variants of genes encoding urate transporters was limited to the transcription regions and exon-intron boundaries.

## Conclusion

The *ABCG2* gene is a well-established hyperuricemia/gout risk locus. In this work, we present the first study of ABCG2 allelic variants in a pediatric-onset hyperuricemia and gout cohort. The high frequency of genetic variants, common and rare, among patients with pediatric-onset hyperuricemia and gout needs to be kept in mind during differential diagnostic procedures and during therapy. Further analysis of the progress of asymptomatic hyperuricemia to gout is necessary: our data suggest a high frequency of the dysfunctional p.Q141K variant in both pediatric-onset subgroups (42.9% in gout, 35.3% in hyperuricemic) compared with adult gout onset (21.2%) and normouricemic controls (8.5%).

When working with patients, genetic data can contribute to more accurate disease prognoses, help personalized lifestyle advice, and improve therapy (urate-lowering therapy choice). The benefits of early initiation of urate-lowering therapy in pediatric-onset patients with a strong genetic risk require careful analysis. Additionally, a discussion regarding the value of a personalized approach to the management of hyperuricemia in clinical practice is necessary.

## Abbreviations

BMI: Body mass index
FE-U: Excretion fraction of urate
GWAS: Genome-wide association studies
MAF: Minor allele frequencies
SU: Serum urate
